# Kuwait Recommendations on Vaccine Use in People with Inflammatory Rheumatic Diseases

**DOI:** 10.1155/2018/5217461

**Published:** 2018-05-13

**Authors:** Ahmad AlEnizi, Khaled AlSaeid, Adel Alawadhi, Eiman Hasan, Entesar H. Husain, Ahmad AlFadhli, Aqeel Ghanem, Fatemah Abutiban, Yaser Ali, Adeeba Al-Herz, Khuloud Mohammed, Waleed Alkandari, Ali Aldei, Hebah Alhajeri, Ahmad Dehrab, Sawsan Hayat

**Affiliations:** ^1^Al Jahra Hospital, Al Jahra, Kuwait; ^2^Faculty of Medicine, Kuwait University, Kuwait City, Kuwait; ^3^Al Amiri Hospital, Kuwait City, Kuwait; ^4^Department of Paediatrics, Faculty of Medicine, Kuwait University, Kuwait City, Kuwait; ^5^Mubarak Al-Kabeer Hospital, Jabriya, Kuwait; ^6^Al Farwaniya Hospital, Al Farwaniya, Kuwait; ^7^Adan Hospital, Hadiya, Kuwait

## Abstract

People with IRD are at increased risk of infection, and in 2011 EULAR made general recommendations for vaccination in these patients. Global and European perspectives are important, but they cannot accurately reflect the individual situations of patients in different countries and regions. Based on our clinical experience and opinions, we have sought to tailor the original EULAR recommendations to include advice for vaccination with new agents approved in the intervening years—including the new class of targeted synthetic disease-modifying antirheumatic drugs. We have also considered the specific demographic needs of patients in local populations in the Gulf region. The resulting 16 recommendations are grouped into four main categories covering general vaccination guidelines and best-practice for all patients with IRD, followed by a set of recommended vaccines against specific pathogens. The last two categories include recommendations for certain patient subgroups with defined risks and for patients who wish to travel.

## 1. Introduction

Inflammatory rheumatic disease (IRD) is an umbrella term designed to encompass a range of conditions (Box 1). It is well-known that people with IRD are at increased risk of infection [1–6], which is thought to be due to both the underlying immune effects of the disease itself and the use of immunomodulatory antirheumatic therapies. Vaccination is an important way to protect against infection, but the efficacy may be reduced in people with immunosuppression compared to the general population [7].

In 2011, the European League Against Rheumatism (EULAR) published 13 evidence-based recommendations for vaccination in people with IRD [8]. In the intervening years there have been several critical changes in the rheumatology landscape, not least the introduction of a new class of targeted synthetic disease-modifying antirheumatic drugs (tsDMARDs)—the janus kinase (JAK) inhibitors. Tofacitinib, the first in class, has been approved in the US since 2012 and in Kuwait since 2013. This new option may be used as first-line therapy after methotrexate failure or, in later lines of therapy, after biologic failure. As such, it is important to understand the vaccination needs for IRD patients receiving tsDMARDs, as well as biologics. Additionally, not all the vaccines cited in the original EULAR recommendations are available or appropriate in Kuwait or the Arabian Gulf region in general, and there may be different considerations for our patients. With this in mind, we sought to tailor the recommendation to local populations in the Gulf region (Kuwait, Saudi Arabia, United Arab Emirates, Bahrain, Qatar, Oman)—with a particular focus on Kuwait—where typical patient profiles and medical practice may vary from those in Europe.

Our expert group undertook a modified DELPHI process to gain consensus on the applicability of the existing EULAR recommendations for our local patients and to make suggestions for amends and updates. In December 2016, a group of 15 rheumatologists and infectious disease specialists met in Kuwait City. The objectives were to understand the implications and application of vaccines in people with IRD in order to develop this adapted guideline. The resulting recommendations are based on the opinions and clinical experience of the authors.

## 2. Vaccines Introduction and Overview

A vaccine is any preparation used as a preventive inoculation, typically a weakened (live) or killed (nonlive) version of the disease-causing bacteria or virus, or parts thereof such as toxins or surface proteins (Box 2).

Vaccines work on conferring immunity against a specific disease or pathogen by stimulating antibody production and generating immune memory. Adult immunity is very different from that of infants and children, since the immune system naturally senesces with age, which has an impact on acquired immunity and the quality of response to vaccination [9, 10]. Antibody responses to vaccination are weaker and decline faster in the elderly or are immunocompromised compared to young healthy people [10].

In older people, the innate immune response is dampened by the reduced activity of neutrophils and macrophages and may be further hampered by persistent inflammation. In parallel, the adaptive immune system also senesces: there is reduced output of naïve T cells and decreased function of memory T cells; B cells also undergo age-related changes that further aggravate the decline [10].

## 3. Regional Considerations

Global and European perspectives are important, but they cannot accurately reflect the individual situations of patients in different countries and regions. This is particularly true for infectious diseases, which can be endemic, and which are tackled with vaccine recommendations and tendering at a country level. Management practices for IRD vary widely and can be affected by cultural differences, socioeconomics, and lack of local infrastructure, but there are signs that strategies in the Middle East—and the Gulf in particular—are evolving [18]. Clinicians are keen to implement up-to-date treatment recommendations, and this includes protecting our patients from contracting unnecessary disease.

Kuwait occupies an area of just under 18,000 square kilometres at the tip of the Arabian Gulf and has a population of 4 million. The Kuwaiti Ministry of Health (MOH) is the major provider of healthcare, although in recent years there has been an emerging contribution from private healthcare clinics [17].

In our region there are significant risk factors for infectious disease; some are common to other parts of the world, but others are distinctive risk factors [19]. A key consideration is the Hajj, during which over 2 million pilgrims from 160 countries gather in Mecca [20]. During this period, cases of vaccine-preventable pneumonia account for one-third of hospitalisations in Saudi Arabia [21–23].

A further consideration is the unique population dynamics in the region, with high numbers of temporary expatriates, which may have a direct impact on healthcare provision and health-economics—as well as potentially affecting the transmission of infectious disease and lowering vaccine coverage rates. Rates of common vaccine-preventable diseases in Kuwait are given in Table 1.

Traditionally, there are a number of barriers to the implementation of vaccination recommendations in adults [24], not least patient attitudes and poor awareness of the safety and benefits of vaccination in general. Even among healthcare workers, vaccination rates are often suboptimal; survey findings in Kuwait suggest that only two-thirds are vaccinated against influenza, with the most common reasons for nonvaccination being lack of time or awareness and doubts about vaccine efficacy [25]. Even in well-resourced systems such as the UK there is suboptimal uptake of vaccines in people with IRD [26]. The patients most often missed are those under 65 years of age who do not have another disease for which vaccination is recommended or incentivised [26]. Additional barriers specific to the Gulf region are inconsistencies in reimbursement and healthcare systems and policies [19].

## 4. DELPHI Process

PubMed searches using MESH terms for IRD, antirheumatic drugs, and vaccines were made from November 2009 to December 2016 in order to identify new literature that could inform the development of amended recommendations. Only articles in the English language and those about patients aged over 18 were included. Other papers considered relevant could be added by the authors at their discretion.

Eighteen draft recommendations were formulated after the initial searches. In a closed online DELPHI exercise, the experts voted on a scale of 0 to 9 to indicate their agreement with each recommendation statement. The calculated means and standard deviations demonstrated that a positive consensus was achieved on 14 recommendations and neutral consensus on the remainder. After discussion and consultation at the December meeting, two recommendations were discarded and two amended. A final DELPHI vote took place on the 16 revised recommendations, the results of which are presented here.

## 5. Recommendations

The recommendations (Table 2) are grouped into four main categories. The first set covers general vaccination guidelines and best-practice for all patients with IRD, followed by a set of recommended vaccines against specific pathogens. The last two categories include recommendations for certain patient subgroups with defined risks and for patients who wish to travel.

## 6. General Vaccination Guidelines

The following recommendations should be followed in all patients with IRD. They include considerations for vaccine administration and general patient management and care. 

*(1) Vaccination Status (including Varicella and HBsAb) Should Be Assessed in the Initial Investigation of Patients with Inflammatory Rheumatic Disease*. It is important to assess the vaccination profile of each individual patient and to document any reported side effects of previous vaccinations [27]. It is important to know which vaccines have been given in the past in order to prescribe necessary catch-up shots or to identify where there may be contraindications to future vaccination [8]. At the patient's first visit, the rheumatologist should therefore take a careful history, and this should be updated at regular intervals to ensure a complete picture [27].

Screening tests are also critical. Since HBV may be reactivated during antirheumatic therapy [28], all IRD patients scheduled to start treatment with DMARDs or biologic therapies should receive screening for HBV infection, followed by antiviral prophylaxis with oral nucleoside analogue as appropriate [28–30]. HBV reactivation is more often seen in people with positive hepatitis B surface antigen (HBsAg) and positive hepatitis B core antibody (anti-HBc), but it can also occur in individuals with resolved infection as defined by a negative HBsAg and a positive anti‐HBc [31–33].

**Figure 1:**
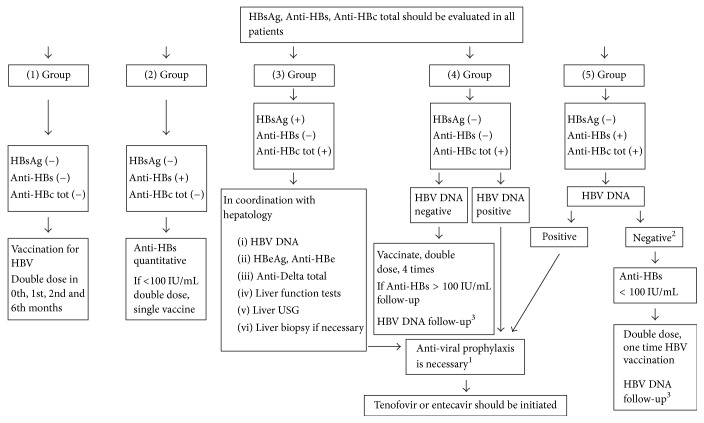
Recommendations for HBV screening and prophylaxis in patients to be administered with bDMARDs, tsDMARDs, or >7.5 mg/day prednisolone [29]. ^1^Treatment period is up until the patient becomes HBsAg negative in patients with chronic hepatitis B (liver disease), and antiviral treatment should continue 6–12 months after immunosuppressive and/or bDMARD treatment is completed in patients without liver disease (12 months for rituximab). ^2^If rituximab is to be administered, antiviral prophylaxis should be given, even if patients are negative for HBV DNA (−). ^3^HBV DNA is repeated once in 1–6 months (3 months on average). Figure reproduced with permission from the European Journal of Rheumatology.

We recommend that HBV serology (HBsAg, anti-HBc, and anti-HBs) should be undertaken in the first instance since it provides information on HBV infection status (Figure 1) [30, 34, 35]. 

*(2) Vaccination Should Be Administered 2–4 Weeks before Starting Immunosuppressive Therapy in Patients with Inflammatory Rheumatic Disease and Ideally Only during Stable Disease*. Vaccination status is best checked and updated before the start of immunotherapy; this ensures that viral replication has ended before any potential decline in immune competence that may result from the long-term antirheumatic treatment [5].

It is still the case that no studies have been performed comparing vaccine efficacy and harm in IRD patients with stable or unstable disease. Some small studies looking at vaccine efficacy in people with active disease do not show an impact on safety or increased disease flare [36]. However, for immunogenicity reasons the expert opinion is that vaccines should be administered 4 weeks before the start of the immunosuppressive treatment and during stable disease wherever possible, unless the risks of remaining unvaccinated far outweigh this position. 

*(3) Vaccination of Patients with Inflammatory Rheumatic Disease Should Be Carried Out by the Treating Rheumatologist or in Collaboration with Public Health Physicians.* All rheumatologists in Kuwait are positioned in secondary or tertiary hospitals, and there is a preventive health department in each major hospital covering all the health districts. The majority of rheumatologists refer patients to the preventive medicine department at their own hospital to receive any recommended vaccinations. In some instances the patient may prefer to get their vaccination in the primary health sector (local polyclinics) in collaboration with and according to instructions by the treating rheumatologist. 

*(4) Live Attenuated Vaccines Should Be Avoided in Immunosuppressed Patients with Inflammatory Rheumatic Disease and Those Receiving Biologic Therapy and Targeted Synthetic DMARDs.* Live attenuated vaccines could lead to severe infections in immunosuppressed IRD patients and should be avoided [27, 36]. However, it is important to understand that this general principle should be balanced against the inherent risk posed by the unvaccinated patient contracting an infection.

Measles, mumps and rubella (MMR), varicella, and herpes zoster live vaccine are exceptions to the rule and may be considered in mildly immunosuppressed patients on an individual basis [8, 37]. Administration of varicella or MMR in children with HIV or after bone transplantation has been documented without subsequent infection [38, 39]. The issue of intravenous immunoglobulin (IVIG) is crucial for paediatric patients. The efficiency of live virus vaccines may decrease if administered less than 2 weeks prior to IVIG (standard or hyperimmunoglobulin) or 1-2 months afterwards. Since IVIG especially suppresses the response to measles vaccine, MMR vaccine should be administered 8–11 months after administration of IVIG. Although the effect of IVIG treatment on varicella vaccine is not fully known, it should be postponed 8 months as with MMR vaccine. If MMR and varicella vaccines have been administered within 14 days before IVIG, they should be repeated after IVIG (8–11 months). Recommendations for the use of live vaccines in patients receiving immunosuppressive therapy are given in Table 3 [40].

In adults with HIV receiving chronic or long-term corticosteroids, live virus zoster vaccine was generally well tolerated and immunogenic [41]. The level of immunosuppression that predisposes an individual to infection is not known. However, when considering any live vaccinations, it is important to emphasise two main considerations. Firstly, the replication capacity of the vaccine, which is, for instance, highest in yellow fever vaccine. Secondly, the risk of complications and exposure to that infection, which is mainly geographical. Temporary discontinuation of immunosuppressive therapy prior to vaccination should be considered and will depend on the half-life of the agent plus the time needed for the immune system to recover its normal state. This is longest for rituximab, although for most immunosuppressive medications this time point has not been clearly defined. 

*(5) Nonlive Attenuated Vaccines Can Be Administered alongside Conventional Synthetic DMARDs and TNFα-Blocking Agents in Patients with Inflammatory Rheumatic Disease, but They Should Ideally Be Administered prior to Starting Biologics or Targeted Synthetic DMARDs Where Possible, Especially for B Cell-Depleting Therapies*. Nonlive attenuated vaccines can be given in patients with IRD alongside DMARDs, glucocorticoids, and TNF inhibitors [27]. Studies looking at the efficacy of this approach mostly show vaccination response comparable to healthy controls [8, 42]. There is reasonable evidence in terms of efficacy also seen with tocilizumab and tofacitinib in regard to flu and pneumococcal vaccines [30, 43]. Of note, methotrexate alone and in combination with TNF inhibitors seems to reduce response to vaccination [27].

An exception to this is anti B-cell antibodies such as rituximab, since the mode of action of these therapies substantially reduces the effect of influenza and pneumococcal vaccination [27, 44, 45]. Abatacept has also been shown to significantly reduce humeral response to flu and pneumococcal and tetanus toxoid if given 2 weeks before vaccination, but when given after vaccination, the response is satisfactory [46–48]. Vaccines should therefore be administered 2–6 weeks before initiating B-cell-depleting therapy [49]. 

*(6) The Household Members of Immunocompromised Patients Can Safely Receive Inactivated Vaccines. Household Members Should Be Up to Date and Vaccinated on Their Recommended Vaccines, Especially Influenza, Varicella, and MMR. Inactivated Polio Vaccine (IPV) Should Be Used instead of the Oral Live Vaccine. Rotavirus Vaccines Should Be Avoided in Household Members of IRD Patients Receiving Biologic Therapy*. There is a concern that immunosuppressed or compromised patients may acquire infections from healthy people around them who are unvaccinated, or who are shedding vaccine-derived viral or bacterial organisms [50].

Adults and children living in the same house as a patient with IRD can safely receive all inactivated (nonlive) vaccines, since viral shedding is unlikely with these preparations. Particular care should be taken to maintain age-appropriate vaccination for all household members and close contacts of people with IRD, especially the siblings of paediatric patients [7, 50–52].

In the rare case that a varicella rash develops in a household member or close contact after varicella or zoster vaccination, there is a risk of transmission to the IRD patient, albeit rare [50, 52]. If blisters develop in the contact at the site of vaccination, the IRD patient should be isolated. Varicella zoster immune globulin could be given prophylactically, and the infected close contact should receive intravenous acyclovir or oral valacyclovir to treat the infection [50].

In general, MMR can be given to individuals with immunocompromised household contacts, who are at increased risk for severe complications from natural measles infection [51, 53]. There is no considerable risk of secondary transmission of measles, mumps, or rubella vaccine viruses from healthy vaccinees caring for or living with immunocompromised contacts [54].

Because of the high risk of viral shedding in faeces and the easy measures that can be taken to prevent severe complications of rotavirus infection, its vaccination should be avoided in the household members of IRD patients receiving bDMARDs or JAK inhibitors. If vaccine is given to any household member, careful measures should be taken to limit the risk of transmission, and all members of the household should wash their hands after changing the infant's diapers—and ideally a parent with IRD should not perform this task for several weeks after vaccination [52].

Around the globe, polio virus infection does still exist, although it has decreased by over 99% since 1988. Today, only three countries (Pakistan, Afghanistan, and Nigeria) have never stopped transmission of polio. In Kuwait, Pakistani residents account for 4% of the general population – representing a significant risk of polio transmission, and hence the importance of measures that should be taken to prevent it, especially given the severe disabling complications of such a condition. The WHO also requires polio vaccination in Kuwait and all GCC countries.

Oral polio vaccine carries a very high risk of viral shedding, which is probably the reason that it is not recommended by EULAR in the households and close contacts of immunocompromised patients. However, with the availability of the inactivated polio vaccine, besides the considerations specific to Kuwait as mentioned above, it is highly recommended that the household members of IRD patients and the IRD patients themselves substitute OPV with IPV when indicated and under the appropriate clinical state as indicated in general vaccinations guidelines number. Rheumatologists should give clear instructions in cooperation with the preventive medicine sectors in this regard. 

*Specific Pathogen Recommendations.* A list of specific vaccine recommendations and timings can be found in Table 4. The rationale for each individual recommendation is outlined below. 

*(7) Nonlive Influenza Vaccination Should Be Strongly Considered for Patients with Inflammatory Rheumatic Disease. The Updated Influenza Vaccine Should Be Given Annually in All Patients with Autoimmune Inflammatory Disease.* People with IRD have an increased risk of dying from pulmonary infection [8]. Nonlive influenza vaccination is efficacious in people with IRD, even alongside concurrent DMARDs or TNF inhibitors [8, 55, 56]. Acceptable vaccine responses are also achieved by patients taking tofacitinib, a new targeted synthetic DMARD [57].

All patients should be vaccinated annually against both seasonal and pandemic influenza strains [58]. The vaccine can be given without the need to withdraw or pause the antirheumatic therapy.

*(8) Conjugate Pneumococcal Vaccination Should Be Strongly Considered for All Patients with Inflammatory Rheumatic Diseases.* Pneumococci are one of the leading causes of pulmonary infections and hospitalisations [8, 19], and pneumonia accounts for a quarter of deaths in people with RA and SLE [59]. Despite this fact, pneumococcal vaccines are infrequently administered to patients with IRD [26].

Pneumococcal vaccination with the 23-valent polysaccharide vaccine (PPV23) induces an adequate to slightly reduced humoral response in patients with IRD. In contrast, methotrexate and rituximab reduce the humoral response following pneumococcal vaccination [8]. Sustained high-dose (>20 mg prednisolone or equivalent for more than 2 weeks) corticosteroid use is also associated with poor vaccine response [59]. US guidelines recommend revaccination after 5 years for those under 65. However, adult response to polysaccharide vaccines relies on immune memory, and as such use of the 13-valent pneumococcal conjugate vaccine (PCV13) may be more appropriate—for which only a single dose is required in adults [58]; that is, only one dose of pneumococcal vaccine is recommended (without booster) according to current knowledge. 

*(9) Patients with Inflammatory Rheumatic Disease Should Receive Tetanus Toxoid Vaccination in accordance with Kuwaiti MOH Recommendations for the General Population. In Case of Major and/or Contaminated Wounds in Patients Who Received Rituximab within the Last 24 Weeks, Passive Immunisation with Tetanus Immunoglobulin Should Be Administered*. The Kuwaiti MOH includes tetanus toxoid vaccination in its schedule of essential vaccination for pregnant women and in all children aged 10–12 years, with a booster at 16–18 years [60].

Passive immunisation with tetanus immunoglobulin may be required in patients who have received rituximab within the past 24 weeks [8, 58]. 

*(10) Hepatitis B Is Endemic in Kuwait. It Is Recommended That All Patients with Inflammatory Rheumatic Diseases Be Screened for Hepatitis B and Vaccinated as Required.* HBV is a major public health problem, with almost one-third of the world population showing serological evidence of current or past infection [33]. The Middle East is defined as a moderate-prevalence region, with infection rates of 2–8%, 2–5% in Kuwait [14]. To address this, there is a designated governmental unit responsible solely for coordinating and carrying out viral hepatitis-related activities, targeted at various populations. All newborns receive the first dose of hepatitis B vaccine prior to discharge [61].

We recommend that all IRD patients be screened for hepatitis B and vaccinated as required if they are found to be at high risk for infection. Specific screening requirements are covered in detail in recommendation (1). 

*(11) Varicella and Zoster Vaccines Can Be Considered in Patients with Inflammatory Rheumatic Disease. Administration Should Be 2–4 Weeks prior to Initiation of Conventional Synthetic DMARDs, High-Dose (>20 mg) Steroids or Biologic Therapies, and Targeted Synthetic DMARDs.* Varicella vaccine is intended for primary prevention. In contrast, the zoster vaccine is designed to reduce reactivation of latent virus residing in sensory ganglia following primary infection, which occurs in 98% of people during childhood [62].

Compared with the general population, people with IRD have an increased risk of reactivation earlier and more frequently than in the general population [8, 62]. Because of the high risk of contracting shingles whilst on immunosuppressive therapy, it is recommended that the vaccine be administered 2–4 weeks prior to the initiation of therapy [58]. As noted previously, in immunosuppressed adults on corticosteroids, live zoster vaccine was generally well tolerated and immunogenic [41, 63]. Efficacy has also been demonstrated with interrupted-dose biologics [64]. At the time of writing, Zostavax is not available in Kuwait, but the group recommend an inclusion in the MOH schedule for this purpose. We recommend its use based on availability for all patients with IRD, especially those above the age of 50. 

*(12) BCG Vaccination Is Not Recommended in Patients with Inflammatory Rheumatic Disease.* Some confusion exists around vaccination against tuberculosis (TB) in people with IRD. Although the incidence of TB is increased in people with IRD, vaccination is not recommended. The reasons for this are twofold: Firstly, Bacillus Calmette-Guérin vaccine (BCG) is a live attenuated vaccine, and as such it is not appropriate for use in people with IRD. Secondly, most cases of active TB are reactivations of earlier latent infections and as such cannot be prevented by vaccination [8]. The efficacy of the BCG vaccine is 50%, with maximum efficacy in preventing TB meningitis and disseminated TB. Of note, the efficacy drops to 20% in immunocompromised HIV patients [65]. 

*For Specific Patient Subgroups.* We highlight two specific groups of patients who require additional consideration with regard to vaccination. 

*(13) Pap Smear Test Screening Should Be Mandated for Sexually Active Females; If Indicated, HPV Vaccination Can Be Given in Both Male and Female Patients with Inflammatory Rheumatic Diseases.* Routine human papilloma virus (HPV) infection is associated with an increased risk of cervical cancer, with significant increase in patients with IRD, particularly SLE [66–68]. Vaccination is therefore recommended for young women in many countries. However, Kuwait has one of the lowest rates of HPV in the world—just 2.3% in women with normal cervical cytology compared to 10% in worldwide studies [16]. Additionally, HPV samples collected in Kuwaiti women are mainly of low-risk types [16]. Thus, we recommend regular Pap smear testing (cervical screening) for all sexually active females, as well as HPV vaccination only where warranted. 

*(14) In Hyposplenic/Asplenic Patients with Inflammatory Rheumatic Disease, Nonlive Influenza, Haemophilus Influenzae b, Conjugate Pneumococcal, and Conjugate Meningococcal ACWY Vaccinations Are Recommended.* Hyposplenic or asplenic patients are at risk of contracting an overwhelming postsplenectomy infection (OPSI) and as such should receive additional vaccination [8].

For Kuwait, we consider the conjugate meningococcal ACWY vaccine to be compulsory in these patients. Recommendations regarding vaccination timing should still be followed. 

*For Patients Who Wish to Travel.* Since they have a suppressed immune system, people with IRD are likely at greater risk of contracting vaccine-preventable diseases when they travel outside of their normal environment. Patients should be encouraged to seek appropriate protection against vaccine-preventable diseases. 

*(15) Patients with Inflammatory Rheumatic Diseases Who Plan to Travel Are Recommended to Receive Their Vaccinations according to General Kuwaiti MOH and CDC Rules, Except for Live Attenuated Vaccines.* International travel is increasingly common for both business and leisure, and modern treatments do not preclude immunocompromised IRD patients from travelling abroad. A pretravel health consultation is therefore important to ensure that patients are adequately protected [69].

Patients with IRD should receive the same vaccinations as recommended for the general population for the country they plan to travel to. Exceptions to this are live attenuated vaccines, including oral typhoid fever and yellow fever vaccines. These should not be given in people with IRD on immunosuppression since these may present a higher risk of causing infections [8]. When live vaccine is indicated, we highly recommend against nonessential travel. Otherwise the biologic treatment should be interrupted if possible for proper vaccinations accordingly giving appropriate timing between treatment break and vaccinations and then resuming the treatment again.

IRD patients not receiving immunosuppression and having a quiescent disease state may receive live attenuated vaccines if indicated for that travel. 

*(16) Patients with Inflammatory Rheumatic Disease Who Wish to Undertake Hajj Should Receive Meningococcal ACWY and Pneumococcal Vaccines within 10 Days to 3 Years prior to Undertaking Hajj and Seasonal Nonlive Influenza Vaccine within 1 Year.* The Hajj is the largest and oldest mass gathering known to mankind. There is a high population density of up to nine people in every square metre. Extreme heat, poorly prepared or stored food, and inadequate pretravel vaccination lead to the spread of many infectious diseases [70].

All patients who wish to undertake the Hajj should receive conjugated versions of the meningococcal and pneumococcal vaccines [19], plus annual influenza. Rheumatologists should be aware that Hajj vaccinations are subject to revision by the Saudi Arabia Hajj regulations. Patients with IRD who plan to undertake Hajj should refer to their rheumatologist in case a new vaccine is required. As a general guide, a schedule for patients with IRD is given in Table 4 [58].

## 7. Discussion

We have sought to tailor the original EULAR recommendations for the Gulf region, taking into consideration our particular climate, geography, and demographics. New classes of agents and new evidence for vaccination have been examined and included. We did not grade our evidence or provide a systematic literature review. Our 16 amended recommendations above are based on the current evidence and our experience as clinicians. There have been reports of flares of IRD after vaccination, but we feel this risk is outweighed by the vast benefits of protecting IRD patients from contracting infections, both for the individual patient and wider society. We encourage clinicians to keep a watchful eye on their IRD patients and to treat any potential flares accordingly.

## Figures and Tables

**Box 1:**
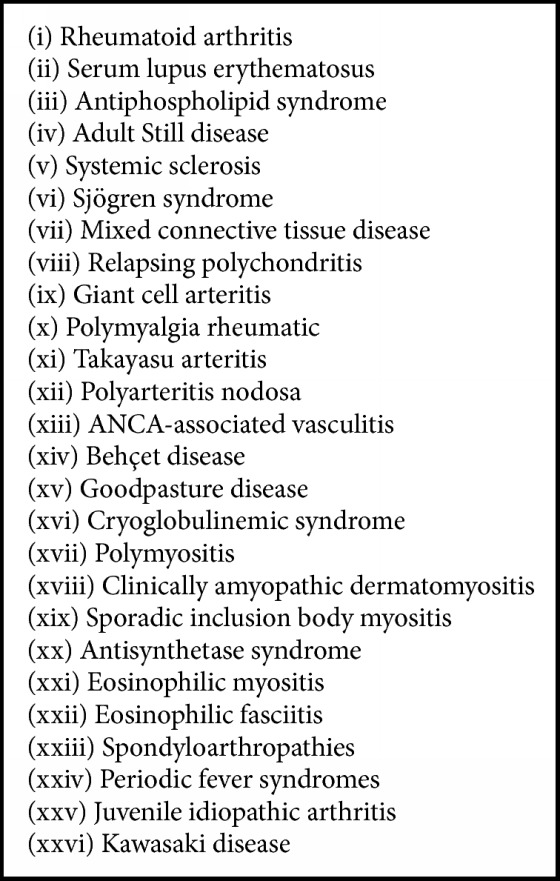
Inflammatory rheumatic diseases [8].

**Box 2:**
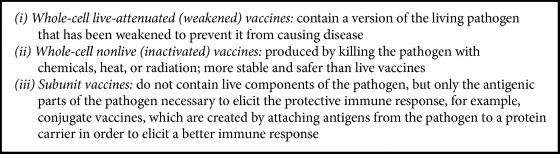
Vaccine overview.

**Table 1:** Rates of vaccine-preventable disease in Kuwait.

Disease	
Pneumococcal disease	5/100,000 annually [11]
Influenza	10–20 per 100,000 population annually [12]
Tetanus^*∗*^	0 [13]
Hepatitis B	2–5% [14]
Herpes zoster	0.55% [15]
Human papilloma virus^*∗∗*^	2.3% [16]
Meningitis	0.5 per 100,000 population annually [17]

^*∗*^There have been no reported cases of tetanus in Kuwait since 1990. ^*∗∗*^In women with normal cervical cytology.

**Table 2:** Recommendations for vaccination in IRD.

Recommendation		Median agreement by DELPHI voting on a scale of 0–9 (IQR)
*General vaccination guidelines*		
1	Vaccination status (including varicella and HBsAb) should be assessed in the initial investigation of patients with inflammatory rheumatic disease	9 (1)
2	Vaccination should be administered 2–4 weeks before starting immunosuppressive therapy in patients with inflammatory rheumatic disease and ideally only during stable disease	9 (0)
3	Vaccination of patients with inflammatory rheumatic disease should be carried out by the treating rheumatologist or in collaboration with public health physicians	9 (2)
4	Live attenuated vaccines should be avoided in immunosuppressed patients with inflammatory rheumatic disease and those receiving biologic therapy and targeted synthetic DMARDs	9 (1)
5	Nonlive attenuated vaccines can be administered alongside conventional synthetic DMARDs and TNF*α*-blocking agents in patients with inflammatory rheumatic disease, but they should ideally be administered prior to starting biologics or targeted synthetic DMARDs where possible, especially for B cell-depleting therapies	9 (1)
6	The household members of immunocompromised patients can safely receive inactivated vaccines. Household members should be up to date and vaccinated on their recommended vaccines, especially influenza, varicella, and MMR. Inactivated polio vaccine (IPV) should be used instead of the oral live vaccine. Rotavirus vaccines should be avoided in household members of IRD patients receiving biologic therapy	9 (1)

*Specific pathogen recommendations*		
7	Nonlive influenza vaccination should be strongly considered for patients with inflammatory rheumatic disease. The updated influenza vaccine should be given annually in all patients with autoimmune inflammatory disease	9 (1)
8	Pneumococcal vaccination should be strongly considered for all patients with inflammatory rheumatic diseases	9 (0)
9	Patients with inflammatory rheumatic disease should receive tetanus toxoid vaccination in accordance with Kuwaiti MOH recommendations for the general population. In case of major and/or contaminated wounds in patients who received rituximab within the last 24 weeks, passive immunisation with tetanus immunoglobulin should be administered	9 (1)
10	Hepatitis B is endemic in Kuwait. It is recommended that all patients with inflammatory rheumatic diseases be screened for hepatitis B and vaccinated as required	9 (1)
11	Varicella and zoster vaccines can be considered in patients with inflammatory rheumatic disease. Administration should be 2–4 weeks prior to initiation of conventional synthetic DMARDs, high-dose (>20 mg) steroids or biologic therapies, and targeted synthetic DMARDs	9 (2)
12	BCG vaccination is not recommended in patients with inflammatory rheumatic disease	8 (2)

*For specific patient subgroups*		
13	Pap smear test screening should be mandated for sexually active females; if indicated, HPV vaccination can be given in both male and female patients with inflammatory rheumatic diseases	9 (1)
14	In hyposplenic or asplenic patients with inflammatory rheumatic disease, nonlive influenza, *Haemophilus influenzae* b, conjugate pneumococcal, and conjugate meningococcal ACWY vaccinations are recommended	9 (0)

*For patients who wish to travel*		
15	Patients with inflammatory rheumatic diseases who plan to travel are recommended to receive their nonlive attenuated vaccines according to general Kuwaiti MOH and CDC rules. Live attenuated vaccines should be avoided in IRD patients on immunosuppression therapy	9 (0)
16	Patients with inflammatory rheumatic disease who wish to undertake Hajj should receive meningococcal ACWY and pneumococcal vaccines within 10 days to 3 years prior to undertaking Hajj and seasonal nonlive influenza vaccine within 1 year	9 (0)

BCG: Bacillus Calmette-Guérin vaccine; CDC: Center for Disease Control; DMARD: disease-modifying antirheumatic drug; HPV: human papilloma virus; IQR: interquartile range; MOH: Ministry of Health; TNF: tumour necrosis factor.

**Table 3:** Live vaccines during immunosuppressive therapy [40].

Therapeutic agent	Herpes zoster/varicella vaccination	Mumps, measles, rubella (MMR), yellow fever vaccination
Low-dose systemic or topical corticosteroids: (i) Short- or long-term daily or alternate-day therapy with <20 mg prednisone or equivalent (ii) Glucocorticosteroid replacement therapy in adrenal insufficiency/topical steroids (airways, skin, ears, or eyes) (iii) Intra-articular, bursal, or tendon injection of steroids Sulfasalazine Hydroxychloroquine	No restrictions	No restrictions

Methotrexate	≤0.4 mg/kg/week (≤20 mg/week): vaccination possible	≤0.4 mg/kg/week (≤20 mg/week): vaccination possible

Azathioprine	≤3.0 mg/kg/day: vaccination possible >3.0 mg/kg/day: contraindication	Contraindication

6-Mercaptopurine	≤1.5 mg/kg/day: vaccination possible >1.5 mg/kg/day: contraindication	Contraindication

Abatacept	Contraindication	Contraindication
Adalimumab		
Anakinra		
Certolizumab		
Cyclosporine A		
Cyclophosphamide		
Etanercept		
Golimumab		
High-dose systemic steroids (≥20 mg per day of prednisone or equivalent for more than 2 weeks)		
Infliximab		
Leflunomide		
Mycophenolate mofetil Rituximab		
Tacrolimus		
Tocilizumab		
Ustekinumab		

Table reproduced with permission from Swiss Medical Weekly—an open access publication of EMH published in accordance with the terms of the Creative Commons Licence Attribution-NonCommercial-NoDerivatives 4.0 International.

**Table 4:** Vaccination schedule in IRD [58].

	Low immunosuppression	High immunosuppression
Influenza	1 dose annually	
Pneumococcal (polysaccharide or conjugate)	1-2 doses	
Tetanus, diphtheria (Td)	Booster every 10 years	
Hepatitis B	3 doses, 0, 1 and 6 months; double doses in high-risk patients initiating bDMARDs or medium/high-dose corticosteroids, depending on serological status	
Hepatitis A	2 doses of vaccine (0 and 6 months)	
Varicella/herpes zoster	Considered in patients with inflammatory rheumatic disease. Administration should be 2–4 weeks prior to initiation of conventional synthetic DMARDs	
Measles, mumps, and rubella (MMR)	Considered in mildly immunosuppressed patients on an individual basis	
Meningococcal (quadrivalent conjugate meningococcal vaccine)	10 days before and up to 3 years before undertaking Hajj Repeated every 5 years if hypo/asplenic state	
*Haemophilus influenza* type B	1 dose	
HPV	2 or 3 doses	

Table reproduced with permission from the European Journal of Rheumatology.
